# Risk factors of gastrointestinal nematode parasite infections in small ruminants kept in smallholder mixed farms in Kenya

**DOI:** 10.1186/1746-6148-3-6

**Published:** 2007-04-20

**Authors:** Agricola Odoi, Joseph M Gathuma, Charles K Gachuiri, Amos Omore

**Affiliations:** 1 Department of Comparative Medicine, The University of Tennessee, Knoxville, TN., USA; 2 Department of Public Health, University of Nairobi, Nairobi, Kenya; 3 Department of Animal Production, University of Nairobi, Nairobi, Kenya; 4 Improving Market Opportunities Theme, International Livestock Research Institute, Nairobi, Kenya

## Abstract

**Background:**

Helminth infections in small ruminants are serious problems in the developing world, particularly where nutrition and sanitation are poor. This study investigated the burden and risk factors of gastrointestinal nematode parasite infections in sheep and goats kept in smallholder mixed farms in the Kenyan Central Highlands. Three hundred and seven small ruminants were sampled from 66 smallholder mixed farms in agro-ecological zones 1 (humid) and 3 (semi-humid) in the Kenyan Central highlands. The farms were visited once a month for eight months during which a health and production survey questionnaire was administered. Fecal samples were collected at each visit from each animal. Fecal egg counts (FEC) were performed using the modified McMaster technique. Associations between potential risk factors and FEC were assessed using 3-level Poisson models fit in SAS using GLIMMIX macro. Correlations among repeated observations were adjusted for using three different correlation structures.

**Results:**

A rise in FEC was observed two months after the onset of rains. Farmer education, age category, de-worming during the preceding month and grazing system were significant predictors of FEC. Additionally, there were significant interactions between grazing system and both de-worming and age category implying that the effect of grazing system is dependent on both de-worming status and age category; and that the effect of de-worming depends on the grazing system. The most important predictors of FEC in the study area were grazing system, de-worming status and education of the farmers.

**Conclusion:**

Since several factors were important predictors of FEC, controlling gastrointestinal helminths of small ruminants in these resource-poor smallholder mixed farms requires a sustainable integrated helminth control strategy that includes adoption of zero-grazing and more farmer education probably through extension services. Achieving improved helminth controls in these resource-poor farming systems offers an opportunity to increase small ruminant productivity and hence has a potential of improving the livelihood of the resource-poor farmers.

## Background

Helminth infections in small ruminants are serious problems of the developing world, particularly where nutrition and sanitation are poor [1,2]. Helminthosis is a primary factor in the reduction of productivity of these animals through mortality and reduced weight gains [3,4]. The problem is greatest in tropical countries with good rainfall [5].

The epidemiology of nematodosis is determined by several factors governed by parasite-host-environment interactions [6-9]. The major risk factors can therefore be broadly classified as parasite factors (including epidemiology of the different species), host factors (genetic resistance, age and physiological status of the animal) and environmental factors (climate, nutrition, stocking density and management). The importance of helminthosis will vary greatly from one year to the next and between geographical locations depending on the prevailing climatic conditions [4]. Moreover, stress, poor nutrition and concurrent disease may be associated with the release of hypobiotic larvae from the dormant state leading to clinical helminthosis. There is also a great variation in resistance between species. While some studies have reported that goats are more susceptible than sheep to a similar challenge, others have reported that sheep usually suffer heavier worm burdens because of the difference in their grazing habits [10,11].

Studies to identify risk factors and control strategies of gastrointestinal nematodes of small ruminants have been performed in many regions of the world [6,8,9,12] and appropriate strategies of helminth control have been designed for specific regions [13,14]. However, helminth control strategies designed for one geo-climatic region and farming system may not necessarily be appropriate for all farming systems and agro-ecological zones due to differences in climatic and management factors. To better identify appropriate control strategies for helminth control of small ruminants in the smallholder systems in Kenya, it is important to identify specific risk factors that are unique to this area and farming system. There is currently very little information on the burden of helminthosis in sheep and goats kept under smallholder mixed farms in the Kenyan Central Highlands. Moreover, specific data on the most important risk factors under these farming practices and agro-ecological zones (AEZs) are also sparse. Therefore, the objective of this study was to investigate the burden and risk factors of gastrointestinal nematodosis in sheep and goats kept on smallholder mixed farms in two AEZs of the Kenyan Central Highlands.

## Results

### Response rates

The initial voluntary enrolment rate was 100% (66 households), all of whom completed the questionnaire used to gather information on farm management, nutrition and helminth control measures. Nine percent (6/66) of those enrolled at the start of the study were lost to follow-up during the study period due to various reasons and therefore the follow-up was completed for 60 farms, representing 91% participation rate.

### Descriptive statistics and simple associations between FEC and potential risk factors

**Figure 1:**
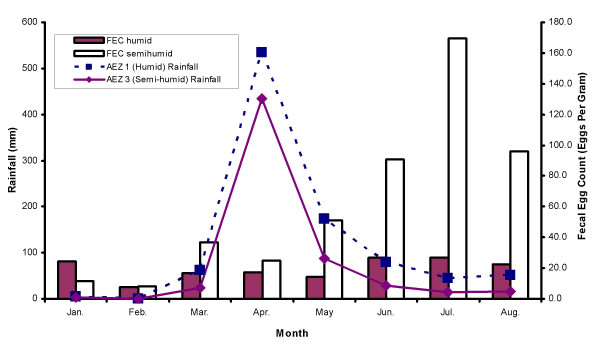
**Relationship between small ruminant fecal egg count and rainfall**. Relationship between fecal egg count (geometric mean) of small ruminants in smallholder farms and rainfall received in two agro-ecological zones (humid and semi-humid) in the Kenyan Central Highlands during the first eight months of 1997.

**Figure 2:**
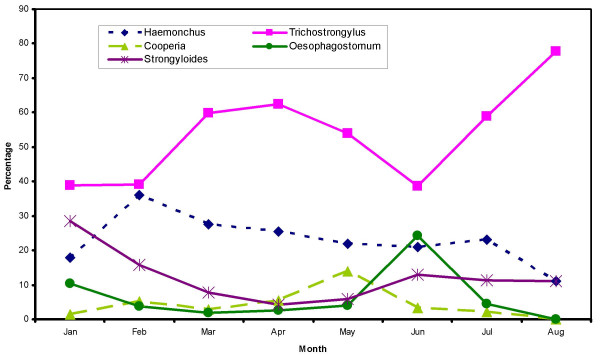
**Genera of gastrointestinal tract nematode larvae cultured from small ruminants in smallholder farms in Kenya**. The figure shows the proportions of different genera of gastrointestinal tract nematode larvae cultured from small ruminants kept on smallholder farms in the Kenyan Central Highlands during the first eight months of 1997.

The fecal egg counts (FECs) were highly variable ranging from 0 to 26100. Over the study period, as many as 49.6% (54.1% in AEZ 1 and 45.9% in AEZ 3) of the recorded FECs were zero. Most (74.6%) of the observed FECs were 500 or fewer eggs per gram. This distribution was similar between the two AEZs (78% in AEZ 1 and 71.5% in AEZ 3). February had the lowest mean FEC (geometric mean = 8.0; range = 0–6,700) and July the highest (geometric mean = 70.0; range 0–10,000). The FEC for AEZ 3 (semi-humid) were consistently higher than those of AEZ 1 (humid) for most of the months, except January and February (Figure 1). The largest difference was observed in July (26.7 in AEZ 1 and 169.6 in AEZ 3). The FEC were relatively low during the months with low rainfall and rose approximately two months after onset of rains reaching their peak in July (Figure 1). The genera of nematodes identified were: *Trichostrongylus *(42.0%), *Haemonchus *(35.8%), *Strongyloides *(12.0%), *Cooperia *(5.5%) and *Oesophagostomum *(4.7%). A summary of the proportions of the different larvae identified over the study period is shown in Figure 2. No significant (P > 0.05) differences in the distribution of nematode genera was observed between the agro-ecological zones. Results of descriptive statistics of categorical independent variables investigated for univariate associations with FEC are given in Table 1. The median age of the farmers was 55 years (range: 25 – 80) while their median number of years of farming experience was 16 years (range: 0 – 60). The farmers reported experiencing land shortage. The median land size was 3 acres with a range of 0 to 16 acres. To supplement grazing, most of the farmers fed maize stover. Regarding farming practices, although most (78%) of the farmers used anthelmintics for helminth control, only 38% sought veterinary advice on anthelmintic use. Moreover, up to 18% of the farmers based their choice of anthelmintics only on the cost price of the drugs. Additionally, most of the farmers practised salvage treatment where by only animals showing obvious clinical disease were treated. Moreover, the treatment was often based on estimation of animal weight which could sometimes lead to under- or over-dosing of the animals depending on the accuracy of the weight estimate made by the farmer.

### Model fit statistics

Based on the observed fit statistics, the first order auto-regressive model had the best fit since it had the smallest values of the AIC and BIC (Table 2). The numbers of correlation parameters as well as the correlation estimates for 1–3 months are shown in Table 2. The correlation estimates show the deficiency of the compound symmetry correlation structure since it assumes the same correlation across all months and does not allow for a reduction in the correlation as the time between measurement gets larger. Although the first order auto-regressive moving average correlation structure allows for this, the decay is much faster than that of the first order auto-regressive model. Based on the best fitting model (first order autoregressive model), there was a reduction in the proportion of variance at the farm level after addition of fixed effects from 10.4% (in the null model without fixed effects) to 7.2% (in the final model). Results of analysis of residuals showed no unduly high residuals.

### Predictors of FEC of small ruminants in smallholder farms in the Kenyan Central Highlands

Education of the farmer (secondary school vs. no secondary school), age category (> = 6 months vs. < 6 months), de-worming during the preceding month (yes/no), and grazing system (free-range, tethering and zero-grazing) were significant predictors of FEC (Table 3). Additionally, there were significant interactions between de-worming during the preceding month and grazing system as well as between grazing system and age category. De-worming during the preceding month is hereafter referred to as de-worming.

The risk ratio for secondary school or higher education was e^-1.476 ^= 0.2 implying that, given the covariates, animals in farms whose owner had at least a secondary school education had 80% [(1-0.2)*100] lower FEC compared to those in farms whose owner had less than secondary school education. Similarly, the risk ratio for AEZ (1 vs. 3) was e^-0.109 ^= 0.9. However, this was not statistically significant (Table 3). The significant interaction terms between de-worming and grazing system implies that the effect of de-worming on FEC depends on the grazing system of the animals and therefore the parameter estimates of the main effects of these variables cannot be interpreted independent of each other. Thus, given the covariates in the model, the risk ratio for de-wormed animals under free-range grazing was e^(-0.599+1.384+1.127) ^= 6.77 implying that the two variables are antagonistic to each other and the benefit of de-worming is off-set by the negative effect of free-range grazing. Similarly, the significant interaction terms between age category and grazing system implies that the effect of age on FEC depends on grazing system. Given the other covariates in the model, the risk ratio for animals that were at least 6 months old under free-range grazing system was e^(-0.563+1.384+0.864) ^= 5.39. Although animal age, free-range grazing, lack of secondary school education of the farmers, and lack of de-worming were all significant predictors of FEC of the small ruminants in the study area, grazing system seems to be the single most important determinant of FEC in small ruminants in smallholder mixed farms in the Central Highlands of Kenya. Surprisingly, the parameter estimate of tethering suggests a protective effect of this grazing system compared to zero-grazing.

## Discussion

This study has identified predictors of FEC or risk factors of nematode parasite infection in small ruminants kept under smallholder mixed farming systems in the Central Highlands of Kenya. The findings are useful in identifying areas for improvement and modification of current helminth control strategies so as to minimize the impact of GIT nematodosis on productivity. The high enrolment (100%) and participation rates (91%) observed in this study is attributed to the fact that the farmers enrolled in this study had already participated in a previous dairy characterisation study and so were farmers interested in participation in research activities. The observed high number of animals with zero fecal egg counts is consistent with the expected FEC distribution (see for instance Polley, 1987) [15] in which only a few animals have high numbers of FEC and most present with no FEC in the fecal samples. The proportions of the genera of nematodes identified in the current study in which *Trichostrongylus *was the most prevalent and *Oesophagostomum *the least, is similar to findings of another study carried out in a semi-arid area of Kenya [7]. However, the order of prevalence reported by a study in Ghana was *Haemonchus*, *Oesophagostomum*, *Trichostrongylus*, and *Cooperia *[16]; while that of a study in South Africa was *Haemonchus*, *Trichostrongylus*, *Ostertagia*, *Cooperia*, and *Oesophagostomum *[17]; and that of a Zimbabwean study was *Haemonchus*, *Oesophagostomum, Trichostrongylus*, *Ostertagia*, *Cooperia*, and *Trichuris *[18]. It therefore seems obvious that differences in prevalent worm genera are dependent on geographical and climatic factors.

The observed rise in FEC approximately 2–3 months after onset of rains is due to the presence of suitable climatic conditions for the development of free-living stages of the nematodes during this time. Although this finding can not be generalized to other years, since only 8 months of a single year were included in this study, it is similar to findings from other studies that FEC are highest during the wet months of the year and start to fall at the onset of the dry season [16]. Contrary to the findings of a study in Germany that found that male sheep had higher FEC than females [19], this study did not find any evidence of differences in FEC based on animal sex. However, similar to reports from other authors [20,21], age was a predictor of FEC in the Central Highlands of Kenya. The protective effect observed in animals 6 months and older is attributable to the delay in development of immunity in animals less than 6 months. Immune response may not be fully developed in sheep before 6–10 months [9,22].

Contrary to reports of other studies that supplementary feeding improves the response of lambs to infection [23-25], this study found no significant association between supplementation of pasture grazing and FEC. This could be attributed to both the quality and quantity of the supplementary feed (mainly maize stover) used by farmers in the current study. Since protein is an essential component in offsetting the parasite induced hypoproteinaemia [25], it is possible that the supplementary feeds used by the farmers were low in protein and so could not significantly effect FEC. This probably resulted in the lack of association observed in this study.  However, analysis of nutritional content of the supplementary feeds was not done in this study because of financial and time constraints. Since GI nematodes result in the loss of endogenous protein [26], feed supplementation is beneficial if the supplementary feed has high protein content. Other studies have reported that sheep infected with *T. colubriformis *increased their protein intake when given a choice between low and high protein diets [27].

The observed positive association between free-range grazing and FEC compared to zero-grazing is due to the increased risk of infection and re-infection in free-range grazed animals compared to their zero-grazed counter-parts. This is in agreement with reports from other authors that under traditional free-range grazing systems there is continuous infection and re-infection from heavily contaminated pastures rendering anthelmintic treatment of limited value compared to the situation under zero-grazing [14]. In zero-grazing production system, helminth control is easier due to decreased risk of exposure to infective larvae. It is therefore not surprising that the parameter estimates for the interaction term between free-range grazing and de-worming revealed a significantly higher risk in de-wormed free-ranged animals compared with non-dewormed zero-grazed animals. Moreover, anthelmintic treatment under free-range system is also expensive due to the need for more treatments per year because of the constant re-infection of treated animals, not to mention the increased chances of development of anthelmintic resistance with the necessary increased use of anthelmintics under the free-range grazing system. It was quite interesting to note that tethering was a protective factor compared with zero-grazing. This may be due to the fact that rotational tethering, in which the animals are not returned to a grazing area until there has been reasonable re-growth of pasture, also provided time for reduction in the number of infective larvae in the pasture resulting in lower risk of infection. Contrary to reports from other studies that there are differences in susceptibilities between sheep and goats, this study did not find significant differences in FEC load between sheep and goats. The reason for this is unclear.

The only helminth control method used in the study area was chemical control and often the treatments were given when clinical helminthoses was evident. The benefits accruing from these salvage treatments were short-lived as treated animals returned to contaminated grazing lands and quickly got re-infected. Additionally, only 38% of the farmers sought veterinary advice on anthelmintic use and as many as 18% based their choice of anthelmintics only on the cost. Although drench-and-move as well as strategic anthelmintic administration have been advocated, their draw back is that they increase the chances of development of anthelmintic resistance [28].

To improve helminth control and productivity in this area, farmers need to integrate management practices aimed at minimizing animal exposure to parasites with reduced reliance on anthelmintics. Therefore, a sustainable integrated helminth control strategy for this area should include adoption of zero-grazing, and effective anthelmintic treatment regimes. An example of an effective anthelmintic treatment strategy that could be adopted in this resource-poor farming system is the FAMACHA^© ^procedure that was developed for resource-poor farming systems in South Africa [13] and has been validated in other countries [29,30]. This system is based on assessment of anemic status of parasitized animals and treating only anemic animals that are succumbing to the effects of helminthoses. Although the drawback of this method is that it assumes that the anemia is solely a result of helminthoses, it has great potential in helminth control in resource-poor farming systems. Compared to conventional strategic anthelmintic treatments where all animals are treated, the FAMACHA^© ^system results in a large proportion of the animals not being treated. If this system were to be used in the study area, its advantage would be three fold: (1) significant cost savings (2) since most of the farmers are already involved in treatment of only clinically parasitized animals, this approach would be readily adoptable (3) untreated animals deposit eggs of anthelmintic-susceptible worms on pasture resulting in maintenance of a reservoir of susceptible larvae *in refugia*. Refugia is the proportion of parasites that are not exposed to a specified parasite control measure, thus escaping selection for resistance [28]. Increasing farmer awareness of alternative and more sustainable control measures could be achieved through education of the farmers of the need for good management practices such as zero-grazing, which are cheaper than over reliance on anthelmintics.

## Conclusion

Based on findings from this study, the most important modifiable predictors of FEC in the study area are grazing system, de-worming status and education of the farmers. Therefore, controlling gastrointestinal helminths of small ruminants in this farming system will require use of an integrated approach that involves adopting non-communal grazing practices, education of farmers and proper use of anthelmintics. We believe that achieving improved helminth control in these farming systems offers an opportunity to increase animal productivity and hence has a potential of improving the livelihood of these resource-poor farmers.

## Methods

### Study area

The study was conducted from January to August 1997 in agro-ecological zones (AEZs) 1 and 3 [31] of the Kenyan Central highlands. The two AEZs were chosen because of their contrasting climatic features. Agro-ecological zone 1 (humid) receives an average rainfall of 1,200 – 2,000 mm per annum whereas the semi-humid AEZ 3 receives 800 – 1,000 mm annually. Both zones receive bimodal rainfall with first rains coming in mid March for AEZ 1 and end of the same month for AEZ 3. The second rains begin in mid and late October in AEZs 1 and 3, respectively. The temperatures are 13.5–16.4°C (AEZ 1) and 15.2–17.4°C (AEZ 3). Altitudes are 2,280–2,550 meters and 1,950–2,070 meters above sea level in AEZs 1 and 3, respectively. The soils are clay and the vegetation is woodland, scrub or grassland.

### Sampling

The sampling frame for this study was derived from a dairy characterisation survey [32]. The above survey sampled households (smallholder mixed farms) from each of the 24 sub-locations in the study area. Survey maps of each of the 24 sub-locations were created in a Geographical Information System (GIS) using ArcInfo GIS software. The survey enumerators, who had previously been trained in the use of the survey instrument, visited their assigned sub-locations, and marked on the sub-location maps the main landmarks. A landmark was defined as any permanent feature such as a trading centre, a school, a church, or a factory [32]. Two pairs of landmarks were then selected at random for each sub-location, and line transects were drawn joining each pair. Sampling was thereafter done following as closely as possible the marked transects. Every 5^th ^household on the left and on the right was included in the survey [32]. The study area had approximately 30,000 households and the dairy characterisation study sampled a total of 365 of these households. These sampled households then provided the sampling frame for the current study which took a random sample of smallholder farms with sheep and/or goats in agro-ecological zones (AEZs) 1 and 3. Smallholder farms were defined as those with less than 20 small ruminants. The first stage of sampling in the current study involved a random selection of nine sub-locations; 5 from AEZ 1 and 4 from AEZ 3. A subset of thirty three farms with sheep and/or goats were then randomly selected from each AEZ. This sampling strategy was deemed to be representative of smallholder mixed farms in the study. All the animals in the sampled farms (households) were included in the study. Sampled farms (households) were visited monthly for 8 months. In order to gather as much data from each animal as possible, animals acquired or borne after the first visit in each farm were not included in the study. Thus, only animals that were present on the farm during the first visit were followed over the study period.

### Data collection

Health reports obtained from the farm owners were recorded for every animal in the study. Data on general farm management, nutrition, and helminth control measures were collected using standard questionnaires that included both closed and semi-closed questions. Data on anthelmintic treatment or de-worming (yes/no) since the previous monthly farm visit were collected from each farm during the monthly visits. The questionnaire is available in English, upon request, from the corresponding author. The questionnaires were pre-tested in three stages: (a) the first stage involved evaluation of the questionnaires by two professors at the University of Nairobi and two researchers at the Kenya Agricultural Research Institute. This part of pre-testing was intended to identify ambiguous questions and to ensure all important issues were covered in the questionnaire. (b) To assess the clarity of the questionnaire, it was administered to 10 randomly selected farmers in the study area and then a follow-up discussion held with each of them to identify and modify questions that were difficult to understand or were ambiguous. (c) A month after the second stage of the pre-testing and after making necessary changes, a third and final part of the pre-testing was performed involving administration of the questionnaire to the 10 farmers in (b) above so as to assess repeatability and to ensure the problem areas identified during the second stage of the pre-testing had been sufficiently addressed. The few questions (< 1%) that were changed significantly after the second stage of the pre-test were not included in the repeatability evaluations since these evaluations would not be valid for such questions. The questionnaire was administered through interviews in *Kikuyu *language. To avoid inter-interviewer variations, only one interviewer administered all the questionnaires for the whole study period. The one-time questionnaire administered at the beginning of the study took approximately 35 minutes. Administration of subsequent monthly questionnaires took less than 5 minutes.

To enhance animal identification, study animals were ear-tagged during the first visit and their identification numbers recorded. Fecal samples were collected from the rectum of each animal during each visit. The samples were placed in plastic fecal bags and transported in a cool box to the laboratory where they were stored at 4°C for periods not exceeding 48 hours prior to processing for parasite egg count. Blinded analyses of fecal egg counts (FEC) were carried out using the McMaster method as modified by MAFF [33]. For genus identification of the nematodes, monthly fecal samples from each farm were pooled and cultured. Nematode larvae (L_3_) were then extracted from the culture using Baermann method and identified. Meteorological data (monthly rainfall totals) were obtained from weather stations nearest the sampled sub-locations.

### Data coding and descriptive analyses

Data from the questionnaires, laboratory analyses and meteorological stations were coded into appropriate variables and entered in a DbaseIV database (DbaseIV, Ashton-Tate, California). The monthly distribution of FECs and weather parameters were displayed graphically using Microsoft Excel software [34]. All statistical analyses were done using SAS [35]. Since both log transformations and computations of geometric means require non-zero values, a factor of "1" was added to all FEC values to ensure that observations with FEC values of zero were included in all computations. Descriptive statistics of farm- animal- and area-level variables were computed as were geometric means of FEC across different animal-, farm- and area-level variables of interest. A list of categorical variables included in these analyses is shown in Table 1.

### Multivariable hierarchical model building

**Figure 3:**
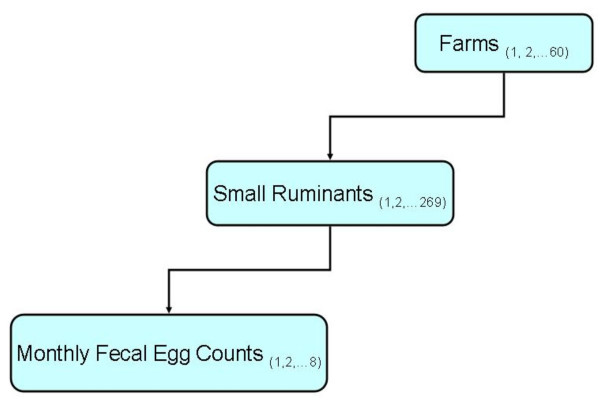
**Hierarchical structure of data used to investigate determinants of fecal egg count**. The figure shows the hierarchical structure of the data used to investigate determinants of fecal egg count of small ruminants kept smallholder mixed farms in two agro-ecological zones (humid and semi-humid) in the Kenyan Central Highlands during the first eight months of 1997.

Univariate associations between log transformed FEC and animal-, farm- and area-level variables were assessed using t-tests (for dichotomous variables) and regression models (for polytomous and continuous variables). A list of the categorical variables assessed for potential univariate association with log transformed FEC are presented in Table 1. There were three continuous variables assessed for potential association with FEC: farmer age, years of farming experience and land size. Variables that had significant univariate associations with FEC were then used to build a multivariable multilevel model. The variable "education of farmer" which originally had 4 levels (no formal education, primary school, secondary school and post-secondary school) was re-categorized into a dichotomous variable by collapsing the four categories into "secondary school or higher" (included "secondary school" and "post-secondary school") and "no secondary school" (included levels "no formal education" and "primary school"). A 3-level hierarchical Poisson model, fit using GLIMMIX SAS macro [36], was used to investigate the relationship between FEC and animal-, farm- and area-level variables that had significant simple (univariate) associations with FEC. The 3-level hierarchy included monthly measures within animals within farms (Figure 3). Note that due to losses to follow-up, the number of animals and farms included in the final analyses were 269 and 60, respectively. Main effects models were fit to the data and all significant main effects were retained. Since the agro-ecological zones were purposively selected, the AEZ variable was not included in the hierarchical structure but was investigated as a fixed effect and forced in the final model. All possible 2-way interaction terms of the main effects were added one at a time to assess their statistical significance. Statistically significant interaction terms were retained in the final competing models.

**Figure 4:**
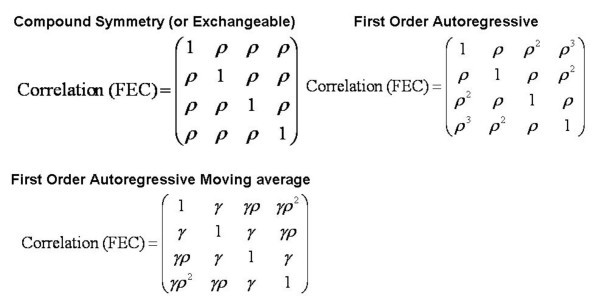
**Correlation structures assessed in the 3-level model used to investigate determinants of fecal egg count**. This figure illustrates correlation structures (representing 4 of the 8 months) assessed in the 3-level model used to investigate determinants of nematode fecal egg count in small ruminants kept in smallholder farms in the Central Highlands of Kenya

The within-animal correlations resulting from the repeated measures within animals were adjusted for in the hierarchical models and the correlation parameters estimated. Three correlation structures (compound symmetry, first order autoregressive, and first order autoregressive moving average correlation structures) were examined (Figure 4) and their appropriateness compared based on how well the resulting models fit the data. The model with the correlation structure that fit the data best was chosen as the best final model. Goodness-of-fit of the three competing models were assessed using -2 log likelihood (deviance), Akaike's Information Criterion (AIC) [37] and the Bayesian Information Criterion (BIC) [38,39]. The goals of the two information criteria are to penalize the assessment of fit by a function of the degrees of freedom and are therefore preferred over deviance since they include a correction of the log likelihood for over-fitting. The information criteria are of the form IC = -2LL + f(K, N) where IC is the information criteria, LL is the maximized log likelihood and f(K, N) is a function of the number of predictors (K) and number of observations (N). For AIC this function corresponds to 2(K+1) and for BIC it is (N-K-1)*ln(N). The model with the lowest value of these criteria was deemed to be the best fitting model. Further model evaluations were carried out by examination of residuals of the best fitting model.

## Authors' contributions

AO (first author) conceived the study, was involved in study design and execution, statistical analysis and writing of the first draft of the manuscript. JMG and CKG participated in study design, and manuscript preparation. AO was involved in study design, coordination of data collection, and manuscript preparation. All authors read and approved the final manuscript.

## Figures and Tables

**Table 1:** Distribution of small ruminant nematode fecal egg counts by potential risk factors.

	**Variable**	**Range**	**FEC**^a^
**Animal-level**			
Age	Less than 6 months	0 – 13000	31.5
	6 months or more	0 – 26100	22.3
De-worming	Yes	0 – 4800	6.7
	No	0 – 26100	24.6
Sex	Male	0 – 26100	24.8
	Female	0 – 18300	25.3
Birth type	Single	0 – 26100	25.7
	Twins	0 – 10000	24.1
Species	Sheep	0 – 26100	25.1
	Goats	0 – 9800	25.2
			
**Farm-level**			
Grazing Supplementation	Yes	0 – 26100	24.4
	No	0 – 13000	26.8
Off-farm grazing	Yes	0 – 11800	35.8
	No	0 – 26100	22.1
Grazing system	Zero-grazing	0 – 26100	34.4
	Tethered	0 – 13000	17.3
	Free-range	0 – 9800	64.7
Anthelmintic brand	Not Known	0 – 1170	29.6
	Albendazole	0 – 2100	13.9
	Levamisole	0 – 13000	38.0
	Levamisole & Oxyclozanite	0 – 9800	12.9
Sex of farmer	Male	0 – 13000	24.2
	Female	0 – 26100	28.2
Education of farmer	No formal education	0 – 11700	68.5
	Primary School	0 – 26100	31.1
	Secondary School	0 – 4800	32.6
	Post-secondary school	0 – 7600	9.1
			
**Area-level**			
AEZ	1	0 – 9500	17.8
	3	0 – 26100	33.5

^The table shows the distribution of fecal egg counts by potential risk factors at the animal-, farm- and area- levels investigated in a study of small ruminants in smallholder farms in the Central Highlands of Kenya^

^a ^geometric mean fecal egg count (Eggs per gram)

**Table 2:** Competing models used to investigate determinants of nematode fecal egg count in small ruminants in Kenya.

	**Compound Symmetry Model**	**First Order Autoregressive Model**	**First Order Autoregressive Moving Average**
**Model Fit Statistics**			
- 2 Log Likelihood	6625.8	6509.3	6577.9
Akaike's Information Criterion (AIC)	6631.8	6515.3	6585.9
Bayesian Information Criterion (BIC)	6638.0	6521.5	6594.2
			
**Correlation Estimates**			
Number of Correlation Parameters	1	1	2
Estimated Correlation			
1 Month	ρ = 0.602	ρ = 0.507	γ = 0.505
2 Months	ρ = 0.602	ρ^2 ^= 0.257	γρ = 0.221
3 Months	ρ = 0.602	ρ^3 ^= 0.130	γρ^2 ^= 0.096

Comparison of repeated measures 3-level models (with different correlation structures) used to investigate determinants of nematode fecal egg count in small ruminants kept in smallholder farms in the Central Highlands of Kenya.

ρ and γ are estimates of different correlation parameters

**Table 3:** Fixed effect estimates of final model used to investigate determinants of nematode fecal egg count in small ruminants in Kenya.

**Fixed Effects**	**Parameter Estimate**	**Standard error of parameter estimate**	**95% Confidence Interval of Estimate**
Secondary School Education (Yes vs. No)	-1.476	0.285	-2.034, -0.918
			
Agro-ecological zone (1 vs. 3)	-0.109	0.210	-0.520, 0.303
			
De-wormed during preceding month (Yes vs. No)	-0.599	0.190	-0.972, -0.226
			
Age (> = 6 months vs. < 6 months)	-0.563	0.212	-0.978, -0.148
			
Grazing System (Reference: Zero-grazing)			
Free-range	1.384	0.678	0.055, 2.713
Tethered	-1.455	0.372	-2.185, -0.726
			
De-worming*Grazing System			
De-wormed*free-range	1.127	0.308	0.523, 1.731
De-wormed*tethered	-1.271	0.348	-1.953, -0.589
			
Age (> = 6 month)*Grazing System			
> = 6 months * free-range	0.864	0.261	0.352, 1.376
> = 6 months * tethered	-1.030	0.588	-2.182, 0.122

Fixed effect estimates and confidence intervals of animal- farm- and area- level variables included in the final first order autoregressive 3-level model used to investigate determinants of high nematode fecal egg count in small ruminants kept in smallholder farms in the Central Highlands of Kenya
